# Nasal Carriage of *Staphylococcus aureus* in Botucatu, Brazil: A Population-Based Survey

**DOI:** 10.1371/journal.pone.0092537

**Published:** 2014-03-24

**Authors:** Fabiana Venegas Pires, Maria de Lourdes Ribeiro de Souza da Cunha, Lígia Maria Abraão, Patrícia Y. F. Martins, Carlos Henrique Camargo, Carlos Magno Castelo Branco Fortaleza

**Affiliations:** 1 Departamento de Doenças Tropicais, Faculdade de Medicina de Botucatu, Universidade Estadual Paulista, Botucatu, Brazil; 2 Departamento de Microbiologia e Imunologia, Instituto de Biociências de Botucatu, Universidade Estadual Paulista, Botucatu, Brazil; Universitätsklinikum Hamburg-Eppendorf, Germany

## Abstract

Recent increases in the incidence and severity of staphylococcal infections renewed interest in studies that assess the burden of asymptomatic carriage of *Staphylococcus aureus* in the community setting. We conducted a population-based survey in the city of Botucatu, Brazil (122,000 inhabitants), in order to identify the prevalence of nasal carriage of *Staphylococcus aureus* (including methicillin-resistant strains). Nasal swabs were obtained from 686 persons over one year of age. Resistance to methicillin was assessed through phenotypic methods, identification of the *mec*A gene and typing of the Staphylococcal Chromosome Cassette *mec* (SCC*mec*). Methicillin-resistant *S. aureus* (MRSA) isolates were characterized using Pulsed-Field Gel Electrophoresis (PFGE), Multilocus Sequence Typing (MLST) and *spa* typing. Polymerase chain reaction was applied to identify genes coding for Panton-Valentine Leukocidin (PVL) in isolates. The prevalence of overall *S. aureus* carriage was 32.7% (95%CI, 29.2%–36.2%). Carriers were significantly younger (mean age, 28.1 versus 36.3 for non-carriers; OR for age, 0.98; 95%CI, 0.97–0.99) and likely to report recent skin infection (OR, 1.85; 95%CI, 1.03–3.34). Carriage of methicillin-resistant *S. aureus* (MRSA) was found in 0.9% of study subjects (95%CI, 0.4%–1.8%). All MRSA isolates harbored SCC*mec* type IV, and belonged to *spa* types t002 or t021, but none among them harbored genes coding for PLV. In MLST, most isolates belonged to clones ST5 or ST1776. However, we found one subject who carried a novel clone, ST2594. Two out of six MRSA carriers had household contacts colonized with isolates similar to theirs. Our study pointed to dissemination of community-associated MRSA among the Brazilian population.

## Introduction

Recent reports point out to an increase in incidence and severity of staphylococcal infections arising in the community [1], [2]. The emergence and worldwide spread of community-associated methicillin-resistant *Staphylococcus aureus* (CA-MRSA) worsened this picture [3]. Most CA-MRSA strains are genetically unrelated to hospital-acquired clones. Also, they have been implicated in the etiology of severe diseases, such as necrotizing pneumonia and skin and soft tissue infection. The severity of CA-MRSA infections has been linked to a major virulence factor, the Panton-Valentine Leukocidin (PVL) [4].

This phenomenon renewed interest in research focusing on the epidemiology of *S. aureus* as a whole and MRSA, both in the community and in healthcare settings. Some studies were directed towards identifying the burden of MRSA infections. Klevens et al. reported that, among 8,987 invasive MRSA infections from 9 sites in the United States (U.S.), 13.7% could be classified as community-associated [5]. From a different perspective, studies from Europe reported MRSA accounting for 6% to 30% of *S. aureus* infections in outpatients [6]. In Asian countries that proportion ranged from 2.5% to 39% [7]. One study conducted in five major African towns, including 542 patients with *S. aureus* infections, identified 86 MRSA isolates, of which 9 were classified as CA-MRSA [8]. Those cases accounted for 11.5% of community-associated *S. aureus* infections, though data may be biased due to criteria for collecting cultures [9].

There is strong evidence of continuous increase in the incidence of CA-MRSA disease during the past decade, at least in the U.S. [10]. Even though data from Europe suggest that the threat posed by CA-MRSA is lower when compared to the U.S., cases of invasive infections are regularly reported in several countries [6], [11], [12]. Reports of CA-MRSA infections in Africa [8] and Asia [7], [13] confirm the worldwide importance of this pathogen.

In order to identify the sources and dynamics of CA-MRSA in the general population, some epidemiological studies addressed the prevalence and determinants of asymptomatic carriage (also termed “colonization”) of *S. aureus* and MRSA in the community setting. Those studies are based on the evidence that colonization represents a reservoir for pathogenic strains and/or a pre-infectious stage [14], [15]. In the U.S., the nasal carriage of *S. aureus* was assessed in 9,622 persons as part of the National Health and Nutrition Examination Survey (NHANES) in 2001–2002. The study found prevalence of 32.4% and 0.8% for *S. aureus* and MRSA, respectively [16], [17]. In the following years, there was a slight decrease in the overall prevalence of *S. aureus* colonization, while the carriage of MRSA almost doubled [18].

Even though extensive research has focused on the global epidemiology of *S. aureus* and MRSA, there are significant gaps of information for regions in Asia, Africa and South America [19]. In Brazil, reports of CA-MRSA infections are scarce, and all data and strains were obtained from patients in university hospitals from highly populated cities [20]–[22].

Our study was designed to fill this gap, providing population-based data about *S. aureus* and MRSA colonization in a small city in inner Brazil. We also aimed to identify factors associated to colonization and the molecular epidemiology of colonizing strains.

## Materials and Methods

### Ethical issues

This study was conducted according to the principles expressed in the Declaration of Helsinki. It was approved by the reference Committee for Ethics in Research (“Comitê de Ética em Pesquisa” from “Faculdade de Medicina de Botucatu”. City of Botucatu, São Paulo State, Brazil). A written informed consent was obtained from all study subjects or their legal guardians.

### Study design and population

We conducted a cross-sectional study in order to identify the prevalence of carriage *S. aureus* among urban population in the city of Botucatu, Brazil (22°53′09″S, 48°26′42″W). The city is located in São Paulo State, in Southeastern Brazil. It has approximately 122,000 inhabitants, 95% of whom live in urban areas. The survey was conducted during the year 2011.

The sample size was estimated in OpenEpi software (Emory University, Atlanta, GA) using the following parameters: city population, 122,000; anticipated *S. aureus* prevalence, 32.4%; confidence limits, 5%; design effect, 2. We estimated the inclusion of 672 subjects. The sample size was later extended for 686, for operational reasons.

The sample was stratified for age and gender, according to the 2010 census data. Subjects with less than one year of age were excluded. Households and subjects from the urban area of Botucatu were selected from an updated version of the database used in the São Paulo State Health Survey [23].

### Collection of swabs and microbiologic methods

Nasal swabs were obtained from the selected subjects. Humidified swabs were inserted in both nares as deeply as possible, until the subjects reported discomfort. Specimens were transported in Stuart medium and cultured in Baird Parker agar. Species identification was performed as follows: The isolates obtained were stained by the Gram method for the determination of purity and observation of morphology and specific staining. After confirmation of these characteristics, the strains were submitted to catalase and coagulase tests. Fermentation tests of maltose, trehalose and mannitol were performed to differentiate *S. aureus* from other coagulase-positive staphylococci [24]. The isolates were also submitted to genotypic identification by Polymerase Chain Reaction (PCR), using primers for amplification of chromosomal DNA fragment which is specific for *S. aureus* (442-bp fragment). We followed the protocol described by Martineau et al. [25] and used primers *Sa442*-1 (5′-AAT CTT TGT CGG TAC ACG ATA TTC TTC ACG-3′) and *Sa442*-2 (5′-CGT AAT GAG ATT TCA GTA GAT AAT ACA ACA-3′).

Susceptibility tests followed guidelines from the Clinical Laboratory Standards Institute (CLSI), using disks for oxacillin and cefoxitin [26].

### Molecular detection and characterization of SCC*mec*

For the purposes of our study, *S. aureus* isolates were classified as MRSA when they harbored the *mecA* gene, which is responsible for methicillin-resistance. PCR for detection of *mecA* was performed as described by Murakami et al. [27]. All reactions included positive (*S. aureus* ATCC 33591) and negative (*S. aureus* ATCC 25923) controls. The multiplex-PCR protocol described by Milheiriço et al [28] was used for the characterization of the staphylococcal cassette chromosome *mec* (SCC*mec*).

### Molecular strain typing

All MRSA isolates were submitted to strain typing. For Pulsed-Field Gel Electrophoresis (PFGE), we used a protocol modified from McDoughal et al. [29], based on DNA digestion with the restriction enzyme *Sma*I. The analysis of similarity was performed using the Dice coefficient. Clusters were defined on the basis of similarity values over 80%. Dendrograms were drawn based on Unweighted Pair Group Method Using Arithmetic Averages (UPGMA) in the BioNumerics 6.1 software (Applied Maths, Belgium). Several MRSA isolates originated from different countries were included as controls in the dendrogram.

Multilocus sequence typing (MLST) was subsequently performed, amplifying and sequencing genes *arcC, aroE, glpF, gmk, pta, tpi* and *yqiL*, as described by Enright et al. [30]. Analysis of results focused on the consensus sequence, according to data from *S. aureus* official MLST site (http://saureus.mlst.net).

*spa* typing was performed according to the protocol described by Shopsin et al [31]. PCR was performed using primers 1095F (5′-AGACGATCCTTCGGTGAGC-3′) e 1517R (5′-GCTTTTGCAATGTCATTTACTG-3′). The amplified product was purified and sequenced. The analysis of repetitions was carried out in the software Bionumerics (Applied Maths, Belgium), which assessed (through specific plugin) the database from Ridom Spa Server (http://www.spaserver.ridom.de).

### Detection of genes coding for PVL

All *S. aureus* isolates were tested for the presence of genes coding for PVL (*lukS-PV, lukF-PV*). We used a PCR protocol, as previously described [32].

### Epidemiological analysis

A questionnaire was applied to all study subjects. Several issues were assessed, including demographics, practice of sports, behavioral factors and comorbidities. Subjects were also questioned about infectious diseases, use of antimicrobials and exposure to healthcare settings during the previous year.

All collected data were stored in database in EPI INFO 3.5 (Centers for Disease Control and Prevention, Atlanta, GA, USA) and analyzed using SPSS 19.0 (IBM, Armonk, NY, USA). Analysis was performed separately for two outcomes: (a) overall *S. aureus* carriage and (b) carriage of MRSA.

All data were initially submitted to bivariate analysis. Dichotomous variables were analyzed using the Chi-square or Fisher Exact test. For continuous variables, we used Student's T or the Mann-Whitney U test. Multivariate analysis was performed using logistic regression models. A “change-in-estimate” approach use used for selection of variables [33]. In the first step, logistic regression included all variables with p<0.1 in the bivariate analysis. New models included all the other variables, one at a time. Variables that changed the Odds Ratio of significant factors in more than 10% were kept in the final model. As counterproof, we performed the multivariate analysis using forward and backward strategies for variable selection, and obtained similar results. P<0.05 was used for definition of statistical significance in all steps of statystical analysis.

## Results

The overall prevalence of nasal carriage of *S. aureus* was 32.7% (95%CI, 29.2%–36.2%). The bivariate analysis of factors associated with carriage is presented in **Table 1**. **Table 2** displays the final multivariate logistic regression model. Briefly, persons carrying *S. aureus* were significantly younger than non-carriers, and more likely to report recent skin infection. Genes coding for PVL were found in five out of 224 isolates (2.2%), all of which were methicillin-susceptible. It is worth noting that only one out of five subjects carrying PVL-positive isolates reported recent skin infection. Given the small number of subjects, the incidence of skin infections in the previous year for carriers of PVL-positive (20%) and PVL-negative (11%) isolates was not statistically significant (p = 0.5).

**Table 1:** Bivariate analysis of factors associated with nasal carriage of *Staphylococcus aureus* among persons living in the city of Botucatu, Brazil, alongside with data from six MRSA carriers in that population.

Factors	*S. aureus* (224)	Controls (462)	OR (95%CI)	*P*	MRSA (6)
*Demographic data*					
**Male gender**	**119 (53.3)**	**185 (40.0)**	**1.70 (1.23–2.34)**	**0.001**	3 (50.0)
**Age, mean (range)**	**28.1 (1–90)**	**36.3 (1–87)**	**…**	**<0.001**	43.7 (20–56)
**Student**	**94 (42.0)**	**130 (28.2)**	**1.84 (1.32–2.57)**	**<0.001**	1 (16.7)
Less than fundamental schooling*	106 (47.3)	187 (41.3)	1.23 (0.93–1.76)	0.13	1 (16.7)
Employed	74 (33.0)	178 (38.5)	0.79 (0.56–1.10)	0.16	4 (66.7)
Family income, median (range) in US dollars**	800 (200–6,000)	750 (350–8,000)	…	0.92	700 (300–2,500)
**Child attending a crèche**	**16 (7.1)**	**22 (4.8)**	**1.84 (0.79–2.99)**	**0.02**	0 (0.0)
Military	0 (0.0)	1 (0.2)	0.0 (…)	1.00	0 (0.0)
Has been in a prison	0 (0.0)	1 (0.2)	0.0 (…)	1.00	0 (0.0)
Has been in a nursing home	1 (0.4)	1 (0.2)	2.07 (0.13–33.20)	0.55	0 (0.0)
*Behavioral factors*					
Smoking	20 (8.9)	56 (12.1)	0.71 (0.42–1.22)	0.21	0 (0.0)
Alcoholism***	30 (13.4)	64 (13.9)	0.96 (0.60–1.53)	0.87	0 (0.0)
Use of illegal drugs (inhaled)	0	1 (0.2)	0.0 (…)	1.00	0 (0.0)
Use of illegal drugs (intravenous)	0 (0.0)	2 (0.4)	0.0 (…)	1.00	0 (0.0)
**Regular practice of sports**	**61 (27.2)**	**86 (18.6)**	**1.64 (1.12–2.38)**	**0.01**	
**Use of earrings**	**76 (33.9)**	**206 (44.6)**	**0.64 (0.46–0.89)**	**0.008**	0 (0.0)
Use of piercing	7 (3.1)	24 (5.2)	0.59 (0.25–1.38)	0.22	0 (0.0)
Tatoo	18 (8.0)	47 (10.2)	0.77 (0.44–1.36)	0.37	0 (0.0)
*Comorbidities and health-related factors*					
Heart disease	5 (2.2)	15 (3.2)			0 (0.0)
Lung disease	14 (6.3)	17 (3.7)	1.75 (0.84–3.61)	0.13	1 (16.7)
Renal disease	0 (0.0)	3 (0.6)	0.0 (…)	0.23	0 (0.0)
Central Nervous System Disease	5 (2.2)	13 (2.8)	0.79 (0.28–2.24)	0.66	0 (0.0)
**Diabetes mellitus**	**4 (1.8)**	**13 (2.8)**	**0.35 (0.12–0.95)**	**0.04**	0 (0.0)
Solid malignancy	2 (0.4)	5 (1.1)	0.83 (0.16–3.30)	1.0	0 (0.0)
Pressure ulcer	2 (0.4)	9 (1.9)	0.45 (0.10–2.12)	1.0	0 (0.0)
Recent skin infection****	25 (11.2)	27 (5.8)	2.02 (1.15–3.56)	0.01	2 (33.3)
Recent pneumonia****	3 (1.3)	11 (2.4)	0.56 (0.15–2.02)	0.57	0 (0.0)
Use of antimicrobials****	56 (25.0)	93 (20.1)	1.32 (0.91–1.93)	0.15	2 (33.3)
Use of steroids****	4 (1.8)	6 (1.3)	1.38 (0.39–4.95)	0.74	0 (0.0)
Admission to a hospital****	14 (6.3)	20 (4.3)	1.47 (0.73–2.97)	0.28	0 (0.0)
Surgery****	8 (3.6)	13 (2.8)	1.28 (0.52–3.13)	0.59	0 (0.0)

Note. All data presented in number(%), unless otherwise specified. Statistically significant results presented in boldface. OR, Odds Ratio; CI, Confidence interval.

*Less than eight years of schooling.

** Data represent the family monthly income. Based on November 2012 exchange parameters (1 dollar is approximately equal to 2 “reais” [Brazilian currency]).

***Regular consumption of alcoholic beverages more than two times a week.

****Events from the past year.

**Table 2:** Final model of multivariate analysis of factors associated with nasal carriage of *Staphylococcus aureus* among persons living in the city of Botucatu, Brazil.

Factors	OR (95%CI)	*P*
**Age**	**0.98 (0.97–0.99)**	**0.01**
Male gender	1.26 (0.82–1.96)	0.3
Student	1.11 (0.71–1.75)	0.6
Attends a crèche	1.03 (0.50–2.12)	0.9
Practice of sports	1.26 (0.84–1.89)	0.3
Use of earrings	0.74 (0.47–1.16)	0.2
Diabetes mellitus	0.56 (0.19–1.68)	0.3
**Recent skin infection** *	**1.85 (1.03–3.34)**	**0.04**

Note. Significant results are presented in boldface. OR, Odds Ratio. CI, Confidence interval.

* In the past year.

On the other hand, MRSA isolates were found colonizing the nares of 0.9% of the study subjects (95%CI, 0.4%–1.8%). None of the six MRSA-colonized subjects reported recent exposure to healthcare settings. All their household contacts (15 persons) were screened, and carriage of MRSA was found in two of them.

**Figure 1:**
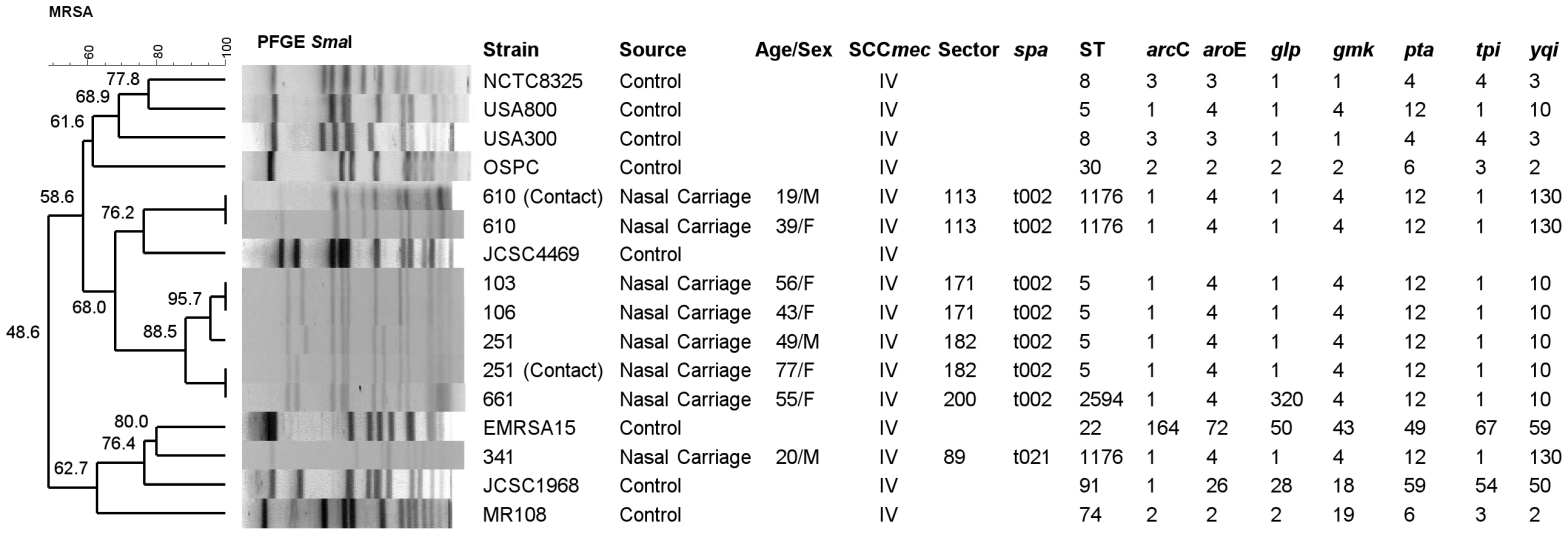
Dendrogram showing Pulsed Field Gel Electrophoresis typing of the study isolates, alongside with results from Multilocus Sequence Typing and *spa* typing. International SCCmec type IV clones are included as controls. Note. Data on age/gender and urban area typing are included. Control strains were kindly provided by Dr. Antonio Carlos Campos Pignatari (Universidade Federal de São Paulo, City of São Paulo, São Paulo State, Brazil) and Dr. Agnes Marie Sá Figueiredo (Universidade Federal do Rio de Janeiro, City of Rio de Janeiro, Rio de Janeiro State, Brazil).

All MRSA isolates harbored SCC*mec* type IV, but none of them carried genes coding for PVL. There was a major PFGE cluster grouping isolates from four out of six MRSA-positive subjects, plus one household contact (**Figure 1**). The isolates in that cluster were characterized as *spa* type t002. In MLST, most belonged to ST5. However, one isolated differed in the allele for the gene *glpF*. The data were submitted to the MSLT homepage (http://saureus.mlst.net), and a novel number was attributed to that allele (320). The isolated was characterized as a novel clone (ST 2594).

The two remaining subjects carried MRSA isolates that presented different PFGE patterns and *spa* types (t002 and t021), but belonged to ST1176. One of them had a household contact colonized with an isolate that was similar to hers in all typing methods.

## Discussion

Several recent studies have assessed *S. aureus* asymptomatic colonization among non-hospitalized persons. Many among them focused on special populations, such as sportsmen [34], military [35], prison inmates [36] or remote communities [37], [38]. Others enrolled specific age groups, such as children [39], [40], [41] and elderly persons [41]. While these studies are of great interest, it is still necessary to estimate the burden of *S. aureus* and MRSA carriage among general population from different countries. Our study aimed to contribute to this issue.

This was the first study to focus *S. aureus* colonization in Brazil using a population-based approach. Our findings point out to a prevalence of both *S. aureus* and MRSA colonization similar to that reported in the 2001–2002 NHANES reports [16], [17] and in minor studies from the U.S. [42], [43]. We found that about one third of the population of Botucatu carries *S. aureus* on their nares. On the other hand, the spread of MRSA in the community is limited. Still, we can estimate more than 1,000 persons carrying MRSA in the city of Botucatu at the time when the study was conducted. This is in contrast with results from two studies that focused on asymptomatic *S. aureus* carriage in Europe. The Tromso Staph and Skin Study enrolled 2,279 subjects, finding prevalence of *S. aureus* nasal colonization of 26.2% for healthcare workers and 26.0% for the general population [44]. Pooled results from Dutch surveys reported colonization in 34.9% of 3,198 children and 27.1% of 3,851 adults over 55 years of age [41]. Despite their sample size, neither studies from Norway or Netherlands found any case of MRSA colonization. At the other end of the epidemiological spectrum in Europe, one study from Malta reported MRSA nasal colonization in 8.8% of 329 healthy individuals [45].

The comparison of our results with data from other countries or continents is difficult, due to differences in the target populations and sites of collection of cultures. Still, it is worth reporting that surveys in African countries found asymptomatic colonization with *S. aureus* and a whole and MRSA in 33% and 9% of 120 persons from the Niger delta (Nigeria) [46] and in 29% and 1% of 500 subjects from rural and semi-urban areas in Lambaréné, Gabon [47]. One study from Queensland (Australia) enrolled 396 patients presenting to general practice and 303 randomly selected volunteers. The prevalence rates of methicillin-susceptible *S. aureus* and MRSA nasal colonization were 28% and 0.7% [48].

Hamdan-Partida et al. [49] investigated 1,243 healthy volunteers from Mexico, finding *S. aureus* and MRSA in 59.8% and 8.6% of study subjects. Those authors followed subjects for 6-years in order to characterize persistent carriage, which occurred in 13.0% of colonized persons. Cultures from nares and throat were performed, and this strategy increased the detection of *S. aureus*. However, the study subjects were selected on the basis of the possibility of long term follow up. Therefore, the sample was not stratified in order to reflect the demographic distribution of Mexican population.

In a study from India, the reported prevalence rates among 1,000 subjects were 22.5% for *S. aureus* and 16.6% for MRSA [50]. A comparison of these results with ours is hindered by the fact that researchers also screened subjects for colonization in body sites other than the nares: namely, forearms and palms. Once again, the approach used for selection of subjects is unclear. Several groups were enrolled, including people from different economic classes, healthcare workers and inpatients. The latter two groups seem overrepresented, so one may doubt the validity of the results as estimates of the colonization burden in the general population.

In our study, we found an inverse association between age and *S. aureus* colonization. This finding has been consistently reported in other studies [16], [18]. Indeed, research that specifically focused on children identified high rates of both *S. aureus* and MRSA colonization [51], [52]. Interestingly, a survey conducted among Pygmies from Gabon identified peek colonization rates among teenagers [38].

In our study, *S. aureus* colonization was associated with the report of skin infections in the past year. In fact, incidence of skin infections during that period was 11.2% for carriers of *S. aureus*. Also, those infections affected two out of six MRSA-colonized subjects. An interesting result is obtained when we reanalyze our database, selecting skin infection as an outcome, and including alternatively colonization with *S. aureus* as a whole or MRSA in multivariate logistic regression models. In this analysis, the previous history of skin infections is independently associated both with the carriage of *S. aureus* (OR, 2.04; 95%CI, 1.11–3.73; p = 0.02) and of MRSA (OR, 9.19; 95%CI, 1.158–53.47; p = 0.01). On the other hand, the presence of PVL genes was not associated to skin infections, possibly due to the small number of isolates harboring those genes.

The association of nasal colonization with *S. aureus* and skin or soft tissue infections was reported as early as the 1960s [53]. In the following decades, studies related colonization with chronic furunculosis and surgical site infections [54], [55], [56], [57]. Based on those results, decolonization with mupirocin has been advised for preventing recurrent skin infections [55], [57].

However, this issue remains controversial [58]. Some studies reported association of *S. aureus* or MRSA colonization with several non-infectious skin diseases [59], [60], [61]. High prevalence of MRSA (of community or healthcare origin) carriage was found in the nares and skin of patients from a dermatology outpatient service [62]. On the other hand, nasal decolonization has failed to prevent recurrent skin infections [63]. Finally, studies focusing on the association of skin infections with colonization of patients and their households reported conflicting results [64], [65], [66].

In regard to our results, as well as those reported in the papers discussed above, one must remember that data obtained from cross-section studies are ambiguous in regard to causality. It is therefore possible that carriage of *S. aureus* led to the development of infection, or that colonization just remained after the infectious syndrome was controlled.

Though limited, our data on MRSA colonization suggest that there are few clones spreading in the community, possibly through social contact. All isolates harbored SCC*mec* type IV, and belonged to Clonal Complex 5 (ST5, ST1176 or ST2594) on MLST. Most of them belonged to the same *spa* type (t002). Finally, a major PFGE cluster comprised isolates from four out of six subjects. Interestingly, two MRSA carriers (subjects #103 and #106) lived on the same street, and their isolates had 100% electrophoretic similarity. Also, two household contacts were colonized with strains similar to those found in their related subjects.

Those findings are noteworthy. Clonal complex 5 is widely spread through America, Europe, Asia and Australia [67]. ST1176 was recently described on clinical cultures from patients admitted to hospitals in the city of São Paulo, 140 miles apart from Botucatu [68]. ST2594 is a novel clone, identified in our survey, and belongs to Clonal complex 5. In summary, our study found evidence of clonal spread on regional and household levels.

The routes of *S. aureus* spread in the community are yet to be elucidated. The same is true for CA-MRSA clones, in spite of extensive research applying methods from classic and molecular epidemiology [19]. It has been suggested that methodological approaches based on social networks may help in filling gaps in our current knowledge. An interesting study addressed this issue focusing on the most basic social network: the household [64]. Analysis of determinants and clonality of *S. aureus* colonization was performed both on individual and aggregate levels. The authors found evidence for transmission within the household, but also for continuous introduction of new strains into households.

Another interesting result concerns PVL. Genes coding for this virulence factor were found in few *S. aureus* isolates, all of which were methicillin-susceptible. The absence of PVL in MRSA isolates colonizing asymptomatic persons is not surprising. In fact, PVL was not an effective marker for CA-MRSA in all studies [69]. Besides, it is expected that PVL-harboring clones are more prevalent among people with active infections.

Our study has several limitations. Most are inherent to the cross-sectional design, which did not allow us to assess the acquisition of *S. aureus* and MRSA over time. Also, new evidence pointed out that relying only on nasal samples may have underestimated the colonization prevalence [49]. The scarcity of subjects with the outcome cast doubt on the validity of the analysis of determinants of MRSA colonization. Finally, authors have questioned the role of nasal colonization as a major issue in CA-MRSA epidemiology [70]. Analyzing data from outbreak reports, they conclude that skin-skin and skin-fomite contact may provide an efficient route for cross-transmission, even in the absence of colonization.

Despite all those limitations, our study has several strengths. First, subjects were selected so as to provide a representative sample of the urban population of the city of Botucatu. Several potential determinants of colonization, including previous contact with healthcare settings, were assessed in the questionnaire. The analysis was extensive, and mixed classical epidemiological and molecular methods to provide the first data on population carriage of *S. aureus* and MRSA in Brazil. Our findings are particularly relevant at a time when CA-MRSA reaches the status of a public health threat. There are lots of issues to be addressed, including policies for screening patients upon admission to hospitals and guidelines for treating suspected staphylococcal infections. Also, surveillance for CA-MRSA infection should be implemented on a nation-wide basis, in order to make sure if data from asymptomatic MRSA carriage correlate with the etiology of skin and soft-tissue infections. It is worth reporting that one adolescent with CA-MRSA cellulitis that started after trauma in a soccer match and progressed to severe sepsis was diagnosed in Bofete, a small town neighboring Botucatu. The isolate belonged to ST5, spa type 311, and harbored PVL genes [71]. As with our results, this occurrence demonstrates the circulation of CA-MRSA in regions distant from major urban centers, and reinforces the necessity of including CA-MRSA in the agenda of public health offices in Brazil.

In conclusion, we found that prevalence rates of *S. aureus* and MRSA colonization in a small city in inner Brazil were similar to those reported in nationwide US surveys. There is evidence of low-level (but consistent) spread CA-MRSA clones, a finding that deserves further investigation. Our results demonstrate the importance of including CA-MRSA among pathogens of interest for public health surveillance at the regional level.
